# Cell line development using the SEFEX system

**DOI:** 10.1186/1753-6561-5-S8-P43

**Published:** 2011-11-22

**Authors:** Benedikt Greulich, Verena Berger, Marika Poppe, Natalie Hepp, Karlheinz Landauer, Andreas Herrmann

**Affiliations:** 1 Celonic AG, Basel, Switzerland; 2 Celonic GmbH, Jülich, Deutschland

## 

Cell lines producing biopharmaceuticals with high yield and high quality in a regulatory compliant environment are a prerequisite for cost effective bioproductions. The development of these production cell lines often includes screening strategies combined with gene amplification and limited dilution experiments, a time consuming process. Especially gene amplification tends to interfere with clonal stability. Limited dilution, especially using a serum-free culture environment is prone to failure and low clone yields.

We present here the SEFEX platform technology for the development of non-amplified high yield production cell lines. The strategy is based on a regulatory compliant method for transfection and single cell cloning using a proprietary, fully tested, CHO-K1 host cell line adapted to chemically defined medium. Fast track cell lines were generated by selection of single cell derived clones within 2.5 months after transfection. 1.0 g/L product concentration were achieved after two rounds of process development using these cell lines. Optimized cell lines were developed based on fast track cell lines employing a second transfection. These cell lines were capable for production of 2.6 g/L during an early phase of process development.

**Figure 1:**
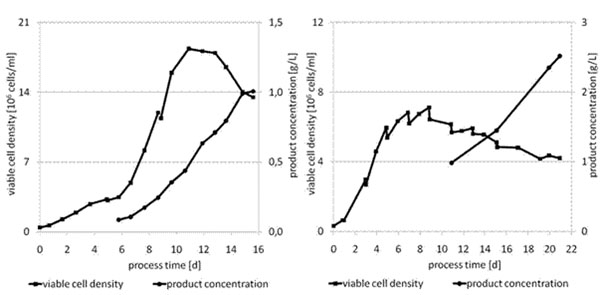
Fed-batch experiments for production of IgG B. The fast-track cell line (left hand graph) was cultivated at 10 L scale after two steps of process development and scale up using chemically defined basal and feed medium. The optimized cell line (right hand graph) was cultivated at 1 L scale after basal media and feed media screening. A chemically defined medium was used in combination with the proprietary feed solution CeloFeed.

Cell line development with the SEFEX technology comprises steps carried out entirely under serum-free conditions including transfection, selection, and single cell cloning. Fast track cell lines were developed within 2.5 months by transfection and selection of stably transfected cells followed by single cell cloning. During single cell cloning, semi-automated photo documentation at the single cell level is used to assure and document clonality in a regulatory compliant manner. Table 1 summarizes cell specific productivity and product concentration in fed-batch processes. More than 1.0 g/L were achieved in a GMP process at 300 L scale after two steps of process development followed by scale-up development. A similar scale-up strategy including GMP production was summarized by Landauer *et al.* in this issue [1]. Nevertheless, production of proteins that are difficult to express like, IgG C in Table 1, suffered from low productivity and low yield. Optimized cell lines, which were obtained by a second serial transfection of the antibody expression vector including regulatory sequences, were suitable to deliver high rates of antibody production. The productivity of IgG C was improved 6-fold to18 pg/c/d. This allowed production of the difficult to express protein at a product concentration of 1.1 g/L in fed-batch mode (Table 1). Other examples of optimized cell lines provided product concentrations of 1.7 g/L without any optimization during cell line development (IgG A, Table 1), or 2.6 g/L after two steps of upstream process development at 1 L bioreactor scale (IgG B, Table 1, Figure 1).

**Table 1:** Cell specific productivity and product concentration obtained with fast track cell lines and optimized cell lines.

	specific productivity [pg/cell/day]		product concentration fed-batch [g/L]	
**Project**	**fast track cell line**	**optimised cell line**	**fast track cell line**	**optimised cell line**

1 (IgG A)	11	36	0.6 ^a^	1.7 ^a^
2 (IgG B)	12	25	1.0 ^b^	2.6 ^c^
3 (IgG C)	3	18	no fed-batch	1.1 ^d^

^a^ Fed-batch experiment during cell line development without any optimization using chemically defined basal medium and proprietary CeloFeed. ^b^ Fed-batch experiment after two steps of process development and scale-up to 10 L using chemically defined basal and feed medium. ^c^ Fed-batch experiment at 1 L bioreactor scale after two steps of optimization using chemically defined basal medium and proprietary CeloFeed. ^d^ Fed-batch experiment at shake flask scale using chemically defined basal medium and proprietary CeloFeed.

Figure 1 shows growth curves of the fast-track and optimized cell line producing IgG B. Optimization of maximum viable cell density was not addressed during upstream process development so far. This optimization is currently ongoing and leaves room for further improvements. Specific productivity of the optimized cell lines varied between 18 and 36 pg/c/d, which was equivalent to a two- to six-fold improvement compared to the fast track cell lines. Productivity was suitable for production of high volume products. Scale-up to 300 L stirred tank and 1000 L wave bioreactor for production of clinical phase II drug product was shown for production cell lines generated with the SEFEX platform technology.

The SEFEX platform technology for the development of non-amplified high yield production cell lines is based on a regulatory compliant, proprietary CHO-K1 host cell line adapted to chemically defined medium. Fast track cell lines, which were available within 2.5 months produced 1.0 g/L product. Optimized cell lines, which were developed based on fast track cell lines, were capable for production of 2.6 g/L in early phase of process development. Using the SEFEX platform technology, all development steps were carried out under entirely serum-free or chemically defined media conditions including transfection, selection, and single cell cloning. Clonality was assured and documented in a regulatory compliant manner using photo documentation of single cells in cell cloning experiments. The serial transfection cell line development strategy described here provides the possibility to develop production cell lines meeting industrial demands employing simplest process development procedures within minimized time frames.
